# Severe Lactic Acidosis in a Patient with B-Cell Lymphoma: A Case Report and Review of the Literature

**DOI:** 10.1155/2009/534561

**Published:** 2010-01-04

**Authors:** Farn Huei Chan, Daniel Carl, Laurel J. Lyckholm

**Affiliations:** ^1^Department of Internal Medicine, Division of Hematology, Oncology and Palliative Care, Virginia Commonwealth University, Richmond, VA 23298, USA; ^2^Department of Internal Medicine, Division of Nephrology, Virginia Commonwealth University, Richmond, VA 23298, USA

## Abstract

Lactic acidosis is commonly observed in clinical situations such as shock and sepsis, as a result of tissue hypoperfusion and hypoxia. Lymphoma and leukemia are among other clinical situations where lactic acidosis has been reported. We present a case of a 59-year-old female with lactic acidosis who was found to have aggressive B-cell lymphoma. There have been 29 cases of lymphoma induced lactic acidosis reported thus far; however all reported cases have abnormal vital signs or concomitant medical conditions that may lead to lactic acidosis. The pathogenesis of malignancy-induced lactic acidosis is not well understood; however associated factors include increased glycolysis, increased lactate production by cancer cells, and decreased hepatic clearance of lactate. When it occurs, lactic acidosis is a poor prognostic sign in these patients. Prompt diagnosis and treatment of underlying lymphoma or leukemia remains the only way to achieve complete resolution of lactic acidosis in these patients.

## 1. Introduction

Lactic acidosis is a common cause of an anion gap acidosis that often carries a significant risk for mortality. Lactic acidosis most commonly results from an imbalance between oxygen delivery and oxygen demand (Type A). However, it can also occur in the absence of a recognizable impairment in systemic oxygen delivery, resulting from impaired oxidative phosphorylation (Type B). Type B lactic acidosis is associated with numerous conditions, including inborn errors of metabolism, drugs and toxins, systemic diseases (i.e., diabetes and sepsis), and less commonly, malignancy. 

Lactic acidosis has been reported in cases of lymphoma and leukemia. The exact pathophysiology and the best way to manage lactic acidosis in this setting remain unclear. We present a case and review the literature relating to lactic acidosis in lymphoma.

## 2. Case Report

A previously healthy 59-year-old high school teacher presented with severe fatigue, generalized weakness, decreased appetite, weight loss, and increased abdominal swelling for 2 months. She reported no fever, night sweats, cough, or urinary symptoms. On physical examination, she appeared cachectic with temporal muscle wasting. Blood pressure was 113/38, heart rate was 134/min, respiratory rate was 22/min, temperature was 98.6°F, and she was not hypoxic on room air. The spleen tip was palpated at 10 cm below the left costal margin. Physical exam findings were otherwise normal. 

**Figure 1:**
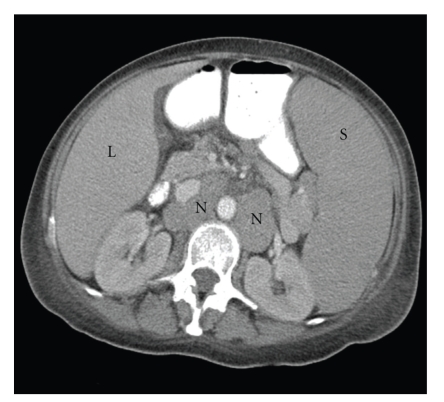
Computed tomography scan of the abdomen showed massive splenomegaly and extensive lymphadenopathy . (L: liver, S: spleen, N: lymphadenopathy).

Laboratory data revealed sodium 134 mmol/L, potassium 4.4 mmol/L, chloride 93 mmol/L, carbon dioxide <10 mmol/L, anion gap >31, blood urea nitrogen (BUN) 25 mg/dL, creatinine 0.6 mg/dL, and glucose 87 mg/dL. Other pertinent labs included leukocyte count 6.4 × 10^9^/L, hemoglobin 4.1 g/dL, platelet count 19 × 10^9^/L, aspartate transaminase (AST) 103 U/L, alanine transaminase (ALT) 15 U/L, alkaline phosphatase 104 U/L, lactate dehydrogenase (LDH) 647 mmol/L (normal range 0.5–2.2 mmol/L), haptoglobin 55 mg/dL, uric acid 13.6 mg/dL, and lactate 16.5 mmol/L. Arterial blood gas revealed a pH of 7.33, a PCO_2_ of 21 mmHg, and PO_2_ of 132 mmHg, with a bicarbonate of 11.2 mmol/L. Peripheral blood smear revealed no blast cells or abnormal leukocytes. Blood cultures were negative. Computed tomography (CT) scan of the abdomen and pelvis revealed massive splenomegaly measuring 25 cm craniocaudally, and extensive lymphadenopathy in the abdomen and pelvis (Figure 1). 

**Figure 2:**
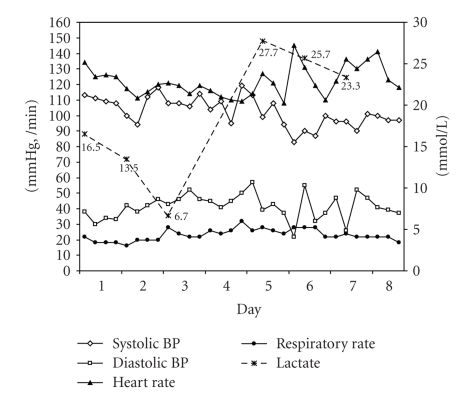
Correlation between lactate level and vital signs.

**Figure 3:**
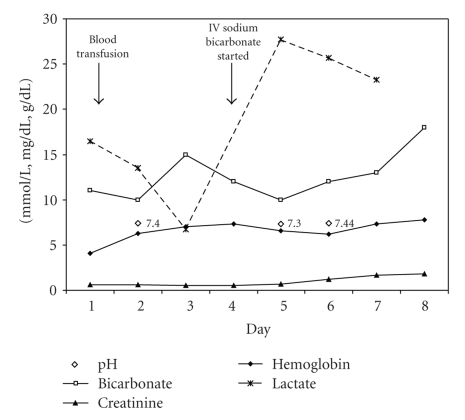
Correlation between lactate level, pH, bicarbonate, creatinine, and hemoglobin.

The patient was aggressively hydrated and received blood transfusions to improve her anemia. However, she remained tachycardic. Throughout her hospital course, her blood pressures remained stable. No source of infection or bleeding was identified. With these measures, lactate improved to 6.7 mmol/L but shortly after that rose to 27.7 mmol/L (Figures 2 and 3). 

While awaiting pathological diagnosis, the patient deteriorated clinically, and on day 4, intravenous corticosteroids were administered for presumed high-grade lymphoma. In addition, a sodium bicarbonate infusion and allopurinol were initiated for tumor lysis syndrome prophylaxis. 

Bone marrow biopsy revealed a diffuse (>50%) infiltrate of cells positive for CD 45 and CD 20, negative for CD 5, CD 10, CD 23, and cyclin D1. GTG-banding studies reveal a very complex karyotype. However, morphologically the cells were not large enough to be called large B-cell lymphoma. The final diagnosis was CD 20-positive B-cell lymphoma. Cyclophosphamide, vincristine, and prednisone were started on day 7. On day 8, she developed respiratory distress with new bilateral pulmonary infiltrates concerning for adult respiratory distress syndrome and became hypotensive requiring vasopressor support. Given the severity of the multiple organ failure, her family decided to withdraw support, and she died on hospital day 12. Autopsy revealed extensive lymphoma as well as diffuse pulmonary, kidney, and esophageal hemorrhage.

## 3. Discussion

Lactate is produced from the anaerobic metabolism of pyruvate, which in turn is generated from glucose via glycolysis. Therefore, lactate production is a surrogate marker of acidosis during a hypoxic state, and not the direct cause. Lactic acidosis is infrequently encountered in malignancies; yet when present portends an extremely poor prognosis [1]. Moreover, the strikingly high mortality associated with lactic acidosis has prompted some oncologists to consider this an oncological emergency [2]. Hematologic malignancies, including acute leukemias and high-grade lymphomas, are the most common neoplastic disorders associated with lactic acidosis. Twenty nine cases of lymphoma induced lactic acidosis have been published in English language journals (Table 1). Seven patients experienced either a partial or complete remission, three of whom subsequently expired from recurrence and there is no long-term followup on an additional two patients. As patients with malignancy induced lactic acidosis are often criticallyill, it is difficult to discern whether the etiology of lactic acidosis is completely a result from the malignancy and not from other potential causes of Type A lactic acidosis (i.e., sepsis, hypotension, hypoxia, etc.). In the patient we present above, she did not have hypoxia or significant hypotension when the lactic acidosis was discovered. The lactic acidosis persisted despite volume repletion, including blood transfusions. Furthermore, we could not identify a source of infection and her lactic acidosis preceded her intensive care visit. However, she was anemic, tachycardic, and tachypneic at presentation. Therefore, it is impossible to conclude there were no additional influences that may have led to Type A lactic acidosis. Upon reviewing the current literature of lymphoma induced lactic acidosis, many cases did have concomitant sepsis, anemia, surgical procedures, or abnormal vital signs (Table 1). Accordingly, every published case that reported vital signs had at least one vital sign indicative of systemic inflammatory response. As the majority of cases to date had concomitant factors that potentially could lead to lactic acidosis, as in the case we present above, the true incidence of lymphoma induced lactic acidosis remains cloudy. 

The pathogenesis of lactic acidosis in lymphoma is incompletely understood and likely multifactorial. Liver metastasis and dysfunction is often cited as a potential cause because of reduced hepatic utilization of lactate via gluconeogenesis [7]. However, lactic acidosis can occur in the absence of liver dysfunction [1, 22, 23]. In fact, Sillos et al. reported 19% of patients with lactic acidosis in the setting of hematological malignancies did not have liver involvement [1]. Furthermore, lactic acidosis is uncommon in patients with cirrhosis or fulminant hepatic failure in the absence of malignancy [24]. Accordingly, although liver dysfunction may contribute to the development of lactic acidosis, it is unlikely to be the sole cause. Another potential mechanism for lactic acidosis is increased glycolytic activity, with a subsequent increase in lactate acid generation, in cancer cells. Further supporting this hypothesis, several cases have been characterized by recurrent hypoglycemia, presumably related to increased glycolysis [7, 9, 12]. Overexpression of type II hexokinase [25], a glycolytic enzyme found in mitochondria, or increased IGF-binding protein (IGFBP) activity, has been implicated in the increased glycolysis in cancer cells [1]. Finally, excessive lactate production may result from highly aggressive tumors that simply outgrow their blood supply [26]. In essence, there is production of lactate from local hypoxia in the absence of any systemic hypoxia or hypoperfusion.

Regardless of etiology, the treatment for lactic acidosis is to discern and correct the underlying mechanism producing the lactate, as well as to ensure adequate oxygen delivery in cases of hypoxia. Accordingly, in malignancy derived lactic acidosis, chemotherapy is the primary treatment modality. As demonstrated in Table 1, the only treatment modality that consistently leads to remission was initiation of chemotherapy. Of the 29 reported cases of lymphoma induced lactic acidosis, only seven went into remission, all of whom received chemotherapy. Of the seven cases who went into remission, the lactate levels normalized in 6 (one case did not report what happened to the lactate levels after chemotherapy). In five of the six cases, resolution of the lactic acidosis occurred as early as 15 hours and up to 3 days after starting chemotherapy. In the remaining case, the lactate level normalized weeks after chemotherapy was introduced; however, did so within 2 days of starting salvage chemotherapy. Thus, it is probably safe to conclude that if lactic acidosis improves after chemotherapy, it occurs in timely manner. Additionally, it is conceivable that prompt resolution of lactic acidosis could be a surrogate marker of inducing remission. 

Both intravenous (IV) bicarbonate and hemodialysis have been used to control the lactic acidosis until chemotherapy can treat the malignancy. The use of IV bicarbonate as an interim treatment of profound acidosis (in either the presence or absence of malignancy) remains a contentious issue [27–30]. As severe acidosis can cause respiratory fatigue and hemodynamic instability, intravenous bicarbonate is often used to attenuate the sequelae of systemic acidosis. However, there are potential side effects from using IV sodium bicarbonate. The most obvious of which are hypervolemia and hypernatremia [31]. Additionally, sodium bicarbonate infusion may actually increase lactic acid production [28]. The rise in lactate levels has been seen in human [32] and animal [33, 34] studies. The decreased oxygen delivery has been postulated to occur from both a reduction in PaO_2_ [35] as well as increased affinity of oxygen to hemoglobin resulting from the rise in systemic pH following IV bicarbonate infusion [36]. The effect of IV bicarbonate on mortality or lactate concentrations in the setting of malignancy induced lactic acidosis has not been directly studied. From our review of the literature, 20 of the 29 cases used IV bicarbonate to treat the acidosis. Two patients received IV bicarbonate without adjunctive chemotherapy, and both died within days. Of the 7 patients who went into remission, 6 were on IV bicarbonate. Although it is unlikely that bicarbonate administration provides any mortality benefit beyond chemotherapy, this has not been directly studied. 

Renal replacement therapy (RRT), including hemodialysis, peritoneal dialysis, or hemofiltration can be used to remove lactate and correct acidosis [37]. Prikis et al. recently reported a case of lymphoma induced lactic acidosis successfully treated with sustained low efficiency dialysis implemented as a temporary measure to correct acidosis and hypervolemia until chemotherapy was initiated [5]. Two other cases of lymphoma induced lactic acidosis have been treated with hemodialysis and chemotherapy, both of whom died within 10 days. As the prognosis of lymphoma induced lactic acidosis is grim, the only hope for remission is starting chemotherapy. Although IV bicarbonate and hemodialysis can be implemented in an attempt to control the acidosis, it must be emphasized that these measures should be viewed as a bridge until the underlying cause is treated.

In conclusion, lactic acidosis is an ominous sign in patients with lymphoma or leukemia. The exact pathogenesis of this condition remains unclear. Prompt diagnosis and early treatment of underlying lymphoma or leukemia is the only way to achieve complete resolution of lactic acidosis in these patients. 

## Figures and Tables

**Table 1:** Summary of all reported cases of lactic acidosis associated with lymphoma. (NR: normal range; BP: blood pressure; RR: respiratory rate; P: pulse; T: temperature.)

Reference	Age	Type of lymphoma	Lactate (mmol/L)	Vitals	Directed treatment of lactic acidosis	Chemo	Outcome	Comments
Friedenberg et al. [3]	75	Follicular	5.4	BP NR P104	None	Yes	Died in 2 days	
Friedenberg et al. [3]	54	T-cell	12	BP 120/50 P 92	None	Yes	Died in 10 weeks	
Friedenberg et al. [3]	54	Diffuse large B-cell	18	BP 96/40 P118	Hemodialysis	Yes	Died in 4 days	Concomitant sepsis (presumed)
He et al. [4]	28	Natural Killer/T-cell	11.2	NR	IV Bicarbonate	Yes	Remission	Died from recurrence after undisclosed period of time
Prikis et al. [5]	65	Large B-cell	18	NR	IV bicarbonate and dialysis	Yes	Remission	Sustained low efficiency dialysis
Dogan et al. [6]	24	Large B-cell	33.0	BP 100/60 P 95 RR18 T 36.7 C	IV Bicarbonate and Hemodialysis	Yes	Died within 10 days	
Glasheen and Sorensen [7]	74	Burkitt's	15.8	NR	None	No	Died in 13 days	
Ohtsubo et al. [8]	77	Mantle Cell	26.3	NR	IV Bicarbonate	Yes	Remission	
DiComite et al. [9]	64	Large B-cell	9.0	NR	IV Bicarbonate	Yes	Died	Unclear time to death
Sillos et al. [1]	18	Large T-cell	15.4	NR	IV bicarbonate	Yes	Remission	Lymphoma recurred and died in 7 months
Thakur et al. [10]	82	Hodgkin's	11.5	NR	IV bicarbonate	No	Died in 3 days	
Yasin and Hartranft [11]	76	Large B-cell	13.1	NR	None	No	Died in 7 days	Presumed ascending cholangitis. Had two exploratory operations
Durig et al. [12]	71	Non-Hodgkin's	17.4	BP 120/60 P120	IV Bicarbonate	Yes	Died in 14 days	
Scheuleer-Holmes et al. [13]	54	Non-Hodgkin's	16.4	BP 160/90 P 92 RR 26 T 37.1 C	IV Bicarbonate	Yes	Died within 3 months	
Caspar and Oelz [14]	74	Non-Hodgkin's	14.8	NR	None	No	Died in 2 days	
Caspar and Oelz [14]	71	Non-Hodgkin's	12.2	BP 165/100 P 100	IV Bicarbonate	Yes	Remission	Died in 6 months
Caspar and Oelz [14]	34	Non-Hodgkin's	23.6	NR	IV Bicarbonate	Yes	Remission	Lactic acidosis occurred after exploratory operation
Doolittle et al. [15]	19	Diffuse Histiocytic	21.8	BP 90/60 P 120 RR 24, Temp 101 F	IV Bicarbonate	Yes	Died in 5 weeks	Blood cultures negative
Doolittle et al. [15]	60	Hodgkin's	16.3	NR	IV Bicarbonate	Yes	Died in 24 days	
Vandermolen et al. [16]	32	Non-Hodgkin's	32	NR	None	Yes	Remission	
Johnson and Whelan [17]	21	Non-Hodgkin's	16.1	NR	IV Bicarbonate	Yes	Died in 14 days	WBC 27,700 and presumed sepsis
Leyden et al. [18]	47	Hodgkin's	20.0	NR	IV Bicarbonate	Yes	Died within 6 days	Pneumonia
Leyden et al. [18]	61	Non-Hodgkin's	46.8	NR	IV Bicarbonate	Yes	Died in one day	Gastrointestinal hemorrhage with hemoglobin of 4.0 g/dL
Nadiminti et al. [19]	30	Hodgkin's	14	BP 120/80 P106	IV bicarbonate	Yes	Died in 4 weeks	
Mintz et al. [20]	65	Histiocytic	21.8	BP 114/66 P 124 RR 32	IV Bicarbonate	No	Died in 4 days	
Mintz et al. [20]	34	Histiocytic	14.2	BP 120/75 P100 T 39C	IV Bicarbonate	Yes	Died in 8 days	
Mintz et al. [20]	60	Histiocytic	5.9	BP 100/65 P 124 RR 30	None	Yes	Died in 3 weeks	
Mintz et al. [20]	58	Histiocytic	4.5	BP 90/60 P 112 RR 50 T 39C	None	Yes	Died in 2 weeks	
Scheerer et al. [21]	47	Hodgkin's	8.8	BP 112/80 P110	IV bicarbonate	Yes	Died in 11 days	
